# Why do International Health Regulations self-assessment capacities (SPAR) scores not predict COVID-19 control outcomes? – analysis of the relationship between SPAR scores and COVID-19 resilience scores in 2021

**DOI:** 10.1186/s12992-025-01111-w

**Published:** 2025-04-15

**Authors:** Fauzi Budi Satria, Feng-Jen Tsai

**Affiliations:** 1 Philosophy Doctor in Medicine Program, Faculty of Medicine, Universitas Sumatera Utara, Medan, Indonesia; 2 Department of Community Medicine, Faculty of Medicine, Universitas Sumatera Utara, Medan, Indonesia; 3 Regional Collaborating Centre, Universitas Sumatera Utara– Singhealth DukeNUS Global Health Institute (USU– SDGHI), Medan, Indonesia; 4 Ph.D. and Master program in Global Health and Health Security, College of Public Health, Taipei Medical University, 250 Wu-Xing Street, Taipei, 110 Taiwan

**Keywords:** International health regulations, Global resilience, State party annual reporting tool (SPAR), Health policy, Pandemic preparedness

## Abstract

**Introduction:**

This study analyses the relationship between SPAR and the COVID-19 resilience score (CRS) in 80 countries in 2021 to achieve its objective.

**Methods:**

We adopted the concept of Bloomberg’s COVID Resilience Ranking to form the CRS, which encompasses three indicators: Reopening Status, COVID Status, and Quality of Life. The average scores of 13 SPAR capacities focused on infectious disease control in 2021 were calculated. Paired t-tests were applied to evaluate the significance of monthly changes in countries’ CRSs. Then, we conducted univariate and multivariate linear regressions to examine the relationship between the SPAR and CRS scores and each CRS indicator.

**Results:**

The CRS in 80 countries fluctuated throughout 2021. Linear regression revealed a significant relationship between countries’ SPAR scores and CRS (B = 0.03, 95% CI = 0.001, 0.06). Among the CRS indicators, the SPAR was significantly associated with only the Quality of Life indicator (B = 0.01, 95% CI = 0.002, 1.52) and not the Reopening Status and COVID Status indicators. An increase in SPAR score, along with an increase in Governmental Effectiveness, was associated with increased CRS (Adjusted R^2^ = 0.52, *p* < 0.05). Moreover, an increase in countries’ SPAR scores was significantly linked to an improvement in people’s Quality of Life (Adjusted R^2^ = 0.37, *p* < 0.05).

**Conclusion:**

The significant relationship between the SPAR and COVID Resilience Scores, particularly the Quality of Life indicator indicates that the lack of accuracy in the ability of the SPAR score to predict COVID-19 control outcomes is attributed to the reliance of the measurement solely on the disease perspective and the limited inclusion of social aspects in the SPAR capacity assessments.

**Clinical trial number:**

Not Applicable

**Supplementary Information:**

The online version contains supplementary material available at 10.1186/s12992-025-01111-w.

## Introduction

The International Health Regulations 2005 (IHR 2005) mandate that member states establish minimum core capacities for pandemic preparedness as an important approach to enhancing global health security [1, 2]. To assess the progress of capacity development, the World Health Organization (WHO) requires member states to annually conduct a self-evaluation of their IHR core capacities, known as the State Party Annual Reporting (SPAR) [1, 2]. Since its implementation in 2007, there has been a gradual increase in member states’ engagement with the IHR [3]. This heightened participation has been demonstrated to aid countries in enhancing their capacity for pandemic preparedness [4–6].

Prior to the COVID-19 pandemic, studies [4, 5] found that this approach was effective and accountable for supporting member states to enhance their ability to address significant public health threats. A study [5] indicated that a 10% increase in SPAR score was associated with a notable decrease of 14–20% in the incidence of transboundary Infectious Disease Threat Events (IDTEs) globally. Furthermore, another study revealed that countries with low SPAR scores face a significantly greater risk of experiencing poor infectious disease outcomes than countries with high average SPAR scores [4]. Based on the assumption that SPAR scores do reflect countries’ capacity, a study in the early phase of the COVID-19 pandemic [7] predicted that countries with higher SPAR scores would experience less severe impacts from the pandemic. However, throughout the COVID-19 pandemic, particularly in the first year, countries with both low and high SPAR scores experienced severe consequences [7, 8].

The devastating impact of the COVID-19 pandemic resulted in it being characterized as a global disaster [9]. Furthermore, in addition to its profound effects on physical health, the pandemic had detrimental effects on the mental and social well-being of most of the global population [10, 11]. As of December 2022, there were more than 600 million confirmed cases of COVID-19 and more than 6 million deaths [11]. Before the pandemic was declared ended on May 5, 2023, it was estimated that by early 2025, COVID-19 would have caused at least 7 million deaths [12]. In addition to health loss [10], reports indicate that the pandemic led to increased inequality, exclusion, discrimination, and unemployment [13]. The COVID-19 pandemic triggered the most severe economic recession since World War II [14], placing significant strain on societies [10, 13, 15]. Such a broad and severe impact of COVID-19 led to concern about countries’ resilience during the pandemic and what it might tell us about preparation for future pandemics.

The United Nations International Strategy for Disaster Reduction (UNISDR) defines resilience as “the ability of an exposed system, community, or society to adapt, bounce back, and recover from the impact of stressors by considering multiple elements, including social, human, physical, financial, and natural resources, in a timely and efficient manner” [16]. This definition emphasizes the significance of considering these elements from a wider perspective, particularly from a social standpoint.

Recognizing the COVID-19 pandemic as a significant disaster that necessitates a focus on countries’ resilience capacity, Bloomberg introduced the COVID Resilience Ranking [17] in November 2020. This ranking system aims to assess countries’ performance in managing the pandemic by evaluating their ability to strike a balance between pandemic control measures and the maintenance of people’s quality of life. Countries with higher resilience rankings demonstrate greater ability to effectively control the spread of the virus while minimizing social and economic disruptions [17]. In contrast to the IHR SPAR score, which mainly focuses on countries’ capacity to detect, assess, and analyze public health epidemics [15], the concept of resilience during COVID-19 expanded from focusing only on health indicators to broader social factors such as Reopening Status and Quality of Life. Throughout the COVID-19 pandemic from the beginning of 2020 to 2023, the continuous debate regarding the policy balance between strict public health measures and economic development and people’s freedom of movement evidenced the importance of involving social concerns in countries’ capacity to respond to the pandemic. Since COVID-19 has been prevalent for over 4 years, much longer than previous pandemics such as SARS, which ended within 6 months, the need for countries to be able to handle the balance between disease control and social concerns has increased. However, the current SPAR tool does not cover such perspectives that might help countries better prepare for the next pandemic.

Since the effects of COVID-19 extended beyond physical health to encompass mental and social well-being [10, 11], it is plausible that the inability of SPAR scores to accurately predict disease control outcomes is due to the lack of ability to balance health measures with social concerns, especially as the pandemic progressed from short-term to medium- and long-term effects.

Therefore, we conducted this study to evaluate the associations between SPAR scores and countries’ resilience during the COVID-19 pandemic. Since resilience is a broader capacity that covers countries’ capacity for health control, SPAR scores should be associated with resilience. Furthermore, with respect to the ability of the IHR to control infectious disease, theoretically, SPAR scores should be significantly associated with health indicators related to resilience. However, because the SPAR does not predict disease control outcomes due to the inability to balance health measures with social concerns, we hypothesized that the SPAR is associated with factors other than health.

## Methods

This ecological study involved an analysis of a total of 80 countries. The inclusion criteria for countries were based on the availability of complete datasets for all variables employed in this study. The focus of this study was to evaluate the COVID Resilience Scores (CRS), a dependent variable, in relation to countries’ SPAR scores related to infectious disease control, serving as the independent variable. Additionally, referring to the Systemic Rapid Assessment (SYSRA) Toolkit [18], we accounted for two confounding variables in this study: Civil Liberties (CL) and Government Effectiveness (GE).

The analysis focused on the second year of the COVID-19 pandemic (2021), a critical phase in which countries had moved beyond the initial emergency response and were transitioning into long-term pandemic management. By this time, vaccination campaigns were underway, public health measures had evolved, and governments were balancing disease control with economic and social considerations.

By examining resilience factors in 2021, this study provides valuable insights into how countries adapted to ongoing challenges, refined their pandemic responses, and integrated public health measures with societal well-being which remain relevant for strengthening preparedness strategies against future health crises.

In this study, certain numerical data variables were transformed into binary categories. This conversion aimed to simplify the description of countries’ characteristics, facilitate scoring, and improve interpretability. The statistical significance threshold was established at *p* < 0.05. All analytical procedures were conducted using R version 4.3.1.

### Countries’ COVID resilience scores (CRS)

Countries’ resilience capacity was assessed using the COVID Resilience Score (CRS), adapted from Bloomberg’s COVID Resilience Rankings [17]. In accordance with Bloomberg’s approach, the CRS has three measurement indicators (Reopening Status, COVID Status, and Quality of Life), each consisting of four measurement components. However, while the CRS retained the three Bloomberg indicators, some components were substituted in order to achieved the study aims (a comparison of indicators is provided in Supplementary Table 1s). In addition, unlike the Bloomberg approach, which analyzes only the fifty-three largest economies [17], CRS analyzes all countries with available data. By using these modified metrics, the CRS represents an attempt to provide a comprehensive assessment of countries’ resilience to the COVID-19 pandemic in 2021.

Under the CRS framework, equal weight proportions are assigned to all three indicators for calculating the CRS scores of these countries. A binary scale (0 or 1) was used to assess the twelve measurement components, and the average score was used as the cutoff point (the scoring system for CRS is provided in Supplementary Table 2s). Consequently, as the CRS encompasses twelve components, the CRS score for each country ranges from 0 (indicating the lowest score) to 12 (indicating the highest score).

### Reopening status

The first indicator in the CRS is the Reopening Status, which aims to evaluate the degree to which countries are advancing toward a return to pre-COVID conditions from perspectives including social activities, economics, business, and tourism [17]. The Reopening Status metric consists of four distinct components: “People Covered by Vaccines” [19], “Lockdown Severity” [20], “Community Immobility” [21], and “Travel Reopening” [22].

“People Covered by Vaccines” [18] was defined as the total number of individuals who received at least one vaccine dose divided by the total population of the respective country. Moreover, “Lockdown Severity” [19] is defined as the stringency index, a composite measure based on nine response indicators encompassing factors such as school closures, workplace closures, and travel bans. This index is rescaled to a value ranging from 0 (indicating openness) to 100 (indicating full closure). The data for these components were sourced from the Our World in Data website.

The “Community Immobility” [20] component is defined as the alteration in the duration of time spent at places of residence relative to a baseline day. The data were collected from both Our World in Data and Google LLC. Moreover, “Travel Reopening” [21] is defined as travel restrictions implemented by governments and includes factors such as country travel restrictions, flight restrictions, the requirement of COVID-19 certificates, quarantine measures, and vaccination coverage. The data of this component were acquired from The Humanitarian Data Exchange (HDX), Office for the Coordination of Humanitarian Affairs (OCHA).

These components were selected to ensure a comprehensive evaluation of reopening progress, encompassing both international and domestic levels. Each individual component within the Reopening Status indicator was assigned a binary score of either 0 or 1 (Table 1s in Supplementary Table 2s). For the “People Covered by Vaccines” and “Travel Reopening” components, a score of 1 is assigned if the value exceeds the cutoff point, while a score of 0 is assigned for values below it. Conversely, for the “Lockdown Severity” and “Community Immobility” components, a score of 1 is given if the value falls below the cutoff point, and a score of 0 is assigned if the value is above it. Consequently, the cumulative score for the indicator of Reopening Status ranges from 0 (indicating a restricted status) to 4 (indicating a lenient status).

### COVID status

The COVID Status indicator aims to determine the severity status of the COVID-19 pandemic in these countries. Four distinct components were utilized to measure this indicator: Reproduction Rate (RR), Case Fatality Rate (CFR), Test per case (TPC), and Positive test Rate (PTR). All of the data for these components were generated from Our World in Data Website [23]. These components were used in the CRS to better illustrate the COVID-19 status of the countries over a one-year timeframe.

The RR was defined as the average number of new infections caused by a single infected individual. The CFR represents the ratio of confirmed deaths to confirmed cases. TPC is the number of tests divided by the number of confirmed cases, while PTR is the number of confirmed cases divided by the number of tests, expressed as a percentage.

Among these 4 components, only the TPC is assigned a score of 1 if its value exceeds the cutoff point and a score of 0 if it is below the cutoff point. Moreover, for the other 3 components, RR, CFR, and PR, a score of 1 is given if their values fall below the cutoff point, and a score of 0 is assigned if their values are above the cutoff point. The total score of the COVID Status indicator for each country ranges from 0 (indicating a severe situation) to 4 (indicating a mild situation).

### Quality of life

The last indicator of CRS is the Quality of Life. In order to assess the Quality of Life, several dimensions and individual perceptions were considered, reflecting the multidimensional nature of the concept, as defined by the WHO. The WHO defines quality of life as an individual’s perception of his or her position in life, considering his or her goals, expectations, standards, and concerns, within the cultural and values systems of his or her environment [24]. Thus, this study incorporates the following components within the Quality of Life indicator in the CRS framework: the Happiness Index [25], the Peace Index [26], annual GDP growth in 2021 [27], and Human Development Index (HDI) [28].

The Happiness Index provides insights into various aspects of well-being. It is a renowned survey that assesses the global happiness levels of countries based on citizens’ self-perceived well-being. This index not only ranks countries worldwide by subjective well-being but also delves into how social, urban, and natural environments collectively influence happiness, providing valuable insights into various dimensions of well-being. The data were sourced from the World Happiness Report 2021 [25].

Annual GDP growth offers an indication of economic performance and development. It is defined as the annual percentage growth rate of GDP at market prices based on constant local currency. These data serve as indicators of a country’s economic performance and development. For this study, we utilized GDP growth data from 2020 to 2021, which were obtained from The World Bank [27].

The Peace Index component’s data were generated from the Global Peace Index 2021 and obtained from the Vision of Humanity website [26]. Providing insights into societal peace and stability, the Peace Index is defined as a composite measure that assesses the peacefulness of countries; it comprises 23 quantitative and qualitative indicators, each weighted on a scale of 1 to 5, where a lower score indicates a higher level of peace.

The final component of the Quality of Life Indicator is the HDI. The HDI reflects the level of human development in each country by measuring three crucial dimensions of human development: a long and healthy life, access to education, and a decent standard of living [29]. Within this indicator, components with values exceeding the predefined cutoff point receive a score of 1, while those falling below the cutoff point receive a score of 0. Consequently, the Quality of Life indicator is assessed on a scale ranging from 0 (indicating poor quality of life) to 4 (indicating fair quality of life) for each country. The data for the HDI component [28] were sourced from the UNDP website.

### Countries’ SPAR scores related to infectious disease control

This study used 2021 SPAR scores for the countries retrieved from the WHO website in January 2022 [30]. In 2021, 15 SPAR capacities were reported by the countries. However, since the focus of this study revolves around infectious disease control capacities, only 13 SPAR capacities directly related to infectious disease control were considered. Capacities related to “Chemical contamination” and “Radiation events” were excluded from the analysis.

The scores assigned to each SPAR capacity ranged from 20 (indicating the lowest score) to 100 (indicating the highest score). A score of 20 signifies that the particular capacity has not yet been established within a country, while a score of 100 indicates the sustainable implementation of the capacity. These scores were utilized to assess the level of infectious disease control capacity in each country, providing insights into people’s readiness and preparedness in combating infectious diseases.

### Confounding variables

Referring to the Systemic Rapid Assessment (SYSRA) [18], this study considered Civil Liberties (CL) [31–33]. and Government Effectiveness (GE) [34] as confounding variables. CL data for this study were collected from the Freedom House website in 2021. It represents the transparency of countries in evaluating their national infectious disease capacity [35], with open communication and information contributing to public supervision and preventing manipulation of assessment outcomes. CL score is rated on a scale from 1 to 7, with 1 representing the highest degree of freedom and 7 the lowest. The score is derived from 15 civil liberties indicators, assessed through a set of specific questions. These indicators are categorized into four subcategories: Freedom of Expression and Belief (4 questions); Associational and Organizational Rights (3 questions); Rule of Law (4 questions); and Personal Autonomy and Individual Rights (4 questions).

Next, GE is a component derived from The Worldwide Governance Indicators (WGI), an extensive framework for assessing governance. Within the WGI, the GE specifically measures the quality of public services, policy formulation, and the implementation of government actions. GE scores range from − 2.50 (indicating the lowest GE) to 2.50 (indicating the highest GE) [34]. This study utilized 2021 GE data obtained from The World Bank website. The inclusion of GE in this study aligns with the findings of previous research that has underscored the pivotal role of good governance and the government’s capacity to prevent and control infectious diseases [36, 37].

### Data analysis

**Fig. 1:**
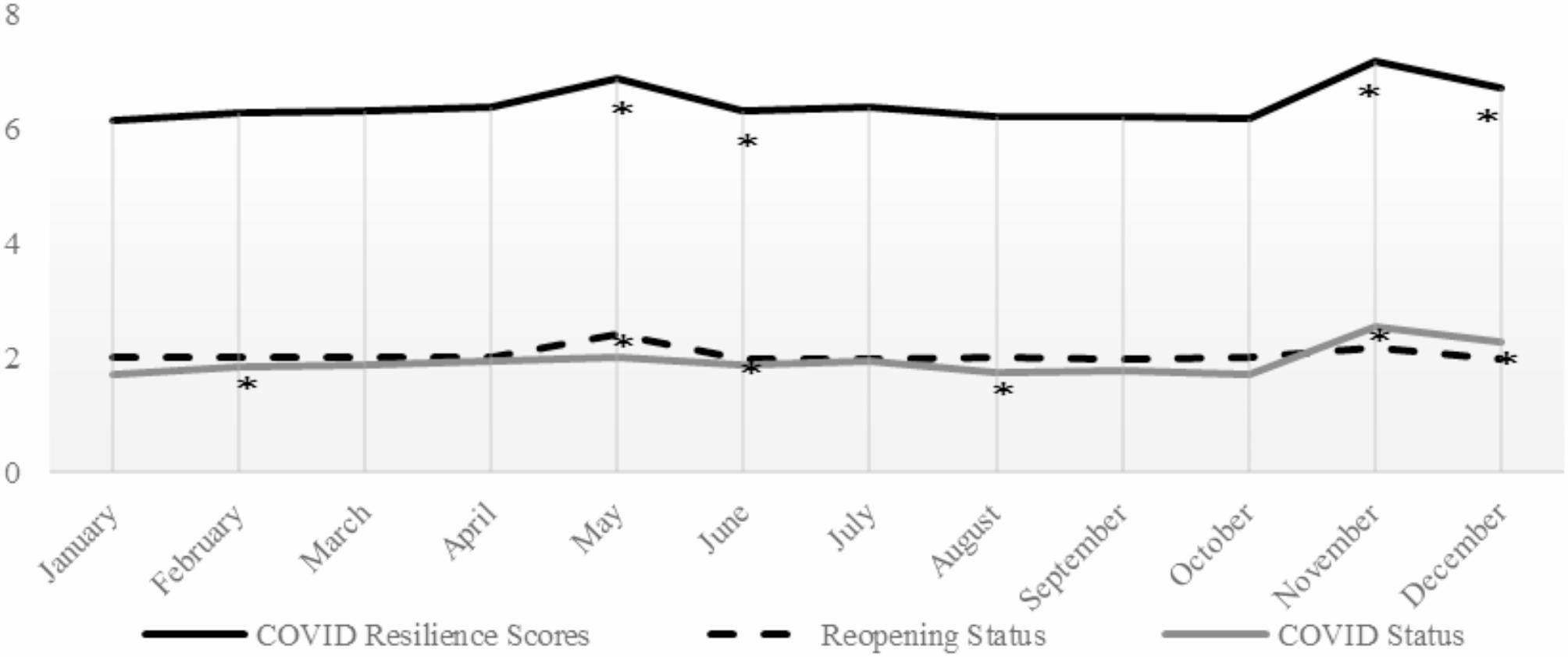
Changes in COVID resilience scores and its indicators in 2021

To evaluate the monthly trend of countries’ CRS and its Reopening and COVID Status indicators, the monthly score of each indicator was calculated and is presented in Fig. 1. For the indicator with daily data, we first calculate the average value of each component from 80 countries every month. These values were subsequently used as cutoff points to classify countries into high- and low-performance groups, with scores of 0 or 1 every month. We then summed the scores of the components of each indicator to obtain the monthly score. This applies to both the Reopening Status and COVID Status. For the indicator with only yearly data, such as Quality of Life, we assumed that the values of each component within this indicator were the same every month throughout the year. We further determined the significance of the monthly changes for Reopening and COVID Status indicators by conducting paired t-tests.

With the cutoff point as the average score, countries were divided into high-SPAR and low-SPAR groups for analysis. The chi-square test was used to compare countries’ characteristics including their income level and geographical region, between the different SPAR and CRS score groups. Then, we conducted univariate and multivariate linear regression analyses to develop Models 1 and 2 to evaluate the associations between countries’ SPAR scores and their CRS. While Model 1 sought to identify associations between countries’ infectious disease control capacities (measured by SPAR scores) and their CRS scores in 2021, Model 2 was developed to ensure internal validity and identify factors that contribute to countries’ CRS in the same year by incorporating confounding variables.

## Results

### Comparison of countries’ characteristics between different SPAR and CRS groups

A comparison of country characteristics between the different SPAR and CRS groups is shown in Table 1. Among the 80 countries analyzed, the majority were high-income countries (HICs) (40%), followed by high-middle-income countries (HMICs) (33.8%), low-middle-income countries (LMICs) (21.3%), and low-income countries (LICs) (5%). European countries constituted 35% of our analyzed countries, followed by Asian regions at 25%, American regions at 21.3%, and African regions at 18.7%. Regarding the CRS, only 3 countries (3.8%) had scores ranging from 9 to 12, while the scores of 6 countries (7.5%) ranged from 0 to 4. The majority of countries (88.7%) had scores ranging from 5 to 8. The geographic distribution of SPAR scores and CRS is uneven, as 63.6% of European countries have CRS scores ranging between 9 and 12, and 40.5% of countries with high SPAR scores are located in Europe. In addition, the chi-square test showed that (Table 1) income level, region, civil liberties and government effectiveness were not significantly different (*p* > 0.05) between the CRS groups. However, all of these countries’ characteristics were significantly different (*p* < 0.05) between the SPAR groups.

**Table 1:** Comparison of countries’ characteristics between CRS and SPAR score groups

	Total (%)	COVID Resilience Score (CRS)				SPAR		
		Score 0 to 4 (28.7%)	Score 5 to 8 (57.5%)	Score 9 to 12 (13.8%)	X^2^	Low (47.5%)	High (52.5%)	X^2^
Income (%)					9.65			22.70*
*LICs*	4 (5.0)	0 (0)	4 (8.7)	0 (0)		3 (7.9)	1 (2.4)	
*LMICs*	17 (21.3)	7 (30.4)	10 (21.7)	0 (0)		14 (36.8)	3 (7.1)	
*HMICs*	27 (33.8)	12 (52.2)	13 (28.3)	2 (18.2)		15 (39.5)	12 (28.6)	
*HICs*	32 (40.0)	4 (17.4)	19 (41.3)	9 (81.8)		6 (15.8)	26 (61.9)	
Region (%)					8.94			15.89*
*Asia*	20 (25.0)	4 (17.4)	12 (26.1)	4 (36.4)		7 (18.4)	13(31.0)	
*Africa*	15 (18.7)	4 (17.4)	11 (23.9)	0 (0)		13 (34.2)	2 (4.8)	
*America*	17 (21.3)	9 (39.1)	8 (17.4)	0 (0)		7 (18.4)	10 (23.8)	
*European*	28 (35.0)	6 (26.1)	15 (32.6)	7 (63.6)		11 (28.9)	17 (40.5)	
*Civil Liberties*					3.75			9.79*
*Score ≥ 4*	30 (37.5)	8 (34.8)	19 (41.3)	3 (27.3)		17 (44.7)	13 (31.0)	
*Score < 4*	50 (62.5)	15 (65.2)	27 (58.7)	8 (72.7)		21 (55.3)	29 (69.0)	
*Government Effectiveness*					3.71			14.92*
*Score ≤ 0*	39 (48.8)	16 (69.6)	21 (45.7)	2 (18.2)		26 (68.4)	13 (31.0)	
*Score > 0*	41 (51.2)	7 (30.4)	25 (54.3)	9 (81.8)		12 (31.6)	29 (69.0)	

* *p* < 0.05

### Changes in COVID resilience scores and its indicators in 2021

The monthly average CRS of the countries is shown in Fig. 1. The CRS exhibited fluctuations from January to December 2021 in 80 countries. The highest CRS scores were recorded in May (6.85 points) and November 2021 (7.15 points), while the lowest was recorded in January 2021 (6.14 points). Paired t-test analysis revealed significant increases in CRS scores (*p* < 0.05) in May (+ 0.47 point from April) and November (+ 0.99 point from October). Conversely, the CRS of these 80 countries significantly decreased (*p* < 0.05) in June (-0.55 point from May) and December (-0.46 point from November) of the same year.

The analysis of the Reopening Status indicator showed that the 80 countries experienced a significant shift toward leniency in May (2.41 points, *p* < 0.05), followed by a subsequent shift toward increased restrictions in June (1.99 points, *p* < 0.05). Furthermore, in comparison to those in the previous month, the COVID Status in these 80 countries were significantly milder in February (1.83 points, *p* < 0.05) and November 2021 (2.54 points, *p* < 0.05), while the COVID Status were more severe in August (1.75 points, *p* < 0.05) and December 2021 (2.26 points, *p* < 0.05).

### Associations between countries’ SPAR and COVID resilience scores in 2021

The results from Model 1 (Table 2) revealed a significant association between the SPAR scores of countries and their CRS scores, including all three indicators. Specifically, an increase in SPAR scores was significantly linked to an increase in CRS (*p* < 0.05) and its three indicators (*p* < 0.05).

Model 2 (Table 3) confirmed the significant association between the SPAR scores of countries and their CRS scores. Model 2 revealed that the SPAR (B = 0.04, *p* < 0.01) and GE (B = 0.90, *p* < 0.01) were factors associated with CRS. An increase in SPAR scores and GE was significantly associated with an increase in CRS (adjusted R^2^ = 0.52, F = 29.11; *p* < 0.01).

**Table 2:** Association between countries’ SPAR and COVID resilience scores in 2021

	B	Std. Error	t	95% CI	F	Adjusted *R*^2^	
**COVID-Resilience Score**							
SPAR*	0.07	0.01	7.11	[0.05, 0.09]	50.49	0.39	
Reopening status							
SPAR*	0.02	0.01	4.44	[0.01, 0.03]	19.69	0.20	
COVID status							
SPAR*	0.02	0.01	3.62	[0.008, 0.03]	13.12	0.14	
Quality of life							
SPAR*	0.03	0.01	5.35	[0.02, 0.04]	28.62	0.27	

* *p* < 0.05

**Table 3:** Factors associated with countries’ COVID resilience scores in 2021

	B	Std. Error	t	95% CI	F	Adjusted R2
**COVID-Resilience Score**					29.11	0.52
SPAR**	0.04	0.01	3.10	[0.01, 0.06]		
*Civil liberties*	-0.03	0.11	-0.27	[-0.25, 0.19]		
GE**	0.90	0.27	3.35	[0.37, 1.44]		
Reopening status					11.49	0.29
*SPAR*	0.01	0.01	1.44	[-0.003, 0.02]		
Civil liberties*	0.17	0.06	3.00	[0.06, 0.28]		
GE*	0.45	0.14	3.36	[0.18, 0.72]		
COVID status					9.08	0.24
*SPAR*	0.01	0.01	1.16	[-0.005, 0.02]		
*Civil liberties*	-0.06	0.06	-1.01	[-0.17, 0.06]		
*GE*	0.25	0.14	1.76	[-0.03, 0.53]		
Quality of life					16.32	0.37
SPAR*	0.02	0.01	2.83	[0.006, 0.04]		
*Civil liberties*	-0.13	0.07	-1.91	[-0.27, 0.006]		
*GE*	0.20	0.17	1.19	[-0.14, 0.54]		

* *p* < 0.05, ** *p* < 0.01

Among the CRS indicators, the findings in Model 2 indicate that countries’ SPAR scores are significantly associated only with the Quality of Life indicator (adjusted R^2^ = 0.37). An increase in countries’ SPAR scores was significantly linked to an increase in their scores in Quality of Life indicator (B = 0.02, 95% CI = 0.006, 0.04).

Moreover, Model 2 revealed that none of the factors included in the model exhibited a significant association with the COVID Status indicator, while factors such as CL (B = 0.17, 95% CI = 0.06, 0.28) and GE (B = 0.45, 95% CI = 0.18, 0.72) were significantly associated with the Reopening Status indicator.

## Discussion

A previous study [38] elucidated that health, as a multidimensional issue, should be approached from various perspectives. According to this study [38], the inability of indices like SPAR to accurately predict outcomes of health measures is attributed to the fact that SPAR is an index assessed solely from a health perspective, employing simplistic metrics such as the number of cases or mortality rate. The resulting predictive inaccuracy is also suggested to stem from SPAR’s failure to incorporate indicators regarding the effectiveness of governance in implementing systems within a country. Despite its inaccuracy in predicting health measure outcomes, the study posits that SPAR serves as an instrument capable of identifying gaps, thereby aiding countries in enhancing pandemic preparedness efforts [38].

Our study found that SPAR scores correlate with COVID Resilience Scores (CRS), suggesting that higher SPAR scores correspond to greater resilience. However, among the three CRS indicators, SPAR is significantly associated only with Quality of Life (QoL) and shows no correlation with COVID Status or Reopening Status. This indicates that while SPAR may reflect certain aspects of societal well-being, it does not predict a country’s outbreak severity or policy decisions on reopening.

Although resilience encompasses more than just QoL, our findings suggest that SPAR aligns primarily with this aspect rather than with COVID-19 prevention outcomes. This supports our initial argument that SPAR is not an effective predictor of infectious disease control. A possible reason for this limitation is that SPAR does not adequately account for the social dimensions that are fundamental to resilience.

The lack of correlation with COVID Status may stem from the fact that SPAR measures preparedness capacity rather than real-time outbreak conditions, which are influenced by external factors such as virus mutations and public behavior. Similarly, reopening decisions involve complex political and economic considerations beyond pandemic preparedness. To improve its relevance in future assessments, additional indicators reflecting governance, public trust, and societal adaptability should be incorporated.

Consistent with earlier studies [38, 39], the results suggest that if the outcome of pandemic control is considered solely from the perspective of physical health, SPAR cannot accurately predict the health outcome. However, when viewed from a social perspective [39], SPAR accurately predict the health outcome of pandemic control through the measurement of the community’s quality of life.

Referring to the profound impact of the COVID-19 pandemic on the quality of life of communities [40], the outcome of pandemic control cannot be adequately measured solely by the number of cases and deaths [38]. This aligns with the WHO’s concept of health, which emphasizes assessing health from physical, mental, and social perspectives [41]. Additionally, measuring disease control outcomes from a social perspective aligns with the “balancing dynamic” concept advocated by the International Health Regulations (IHR) of 2005. This concept [42] posits that disease control should continue to consider the community’s quality of life in a balanced manner.

Our study’s findings, indicating that SPAR scores are correlated with CRS, especially the Quality of Life indicator, suggest that a country’s resilience depends on the quality of life of its population. The better the quality of life, the higher the resilience of countries. In the context of a pandemic, it is mentioned that a resilient country effectively controls the pandemic while maintaining economic growth [15, 16]. Thus, in our view, a country with good resilience is one that can implement the “balancing dynamic” concept in controlling the COVID-19 pandemic.

Balance does not always mean equal [43]. Based on theory of balance in health [44], balance is an approach about how to trade off priorities, make choices and achieve harmony across different cultures and historical periods to achieve health. Therefore, it’s understood that how a country applies this balancing dynamic concept depends on its respective situation. Out of the 80 countries we analyzed, only 8 countries have the same score for each CRS measurement indicator, while the other 72 countries have different scores for each CRS indicator.

This pattern persists even among countries with similar characteristics. For example, the highest CRS score obtained by a country in this study is 10, and only 5 countries achieved this score. These five countries have similar profiles, all having high SPAR scores, with four being High-Income Countries (HICs) and only one being a High-Middle-Income Country (HMIC). Although their CRS scores and characteristics may be considered similar, the scores for each indicator in each country are not the same.

In addition to the SPAR, the Government Effectiveness (GE) score is also a contributing factor to the CRS of countries. A higher SPAR score signifies a higher level of country resilience. Previous studies [45, 46] have emphasized the significance of governance in infectious disease control. The quality of GE is related to how effectively and efficiently countries allocate their resources to disease control efforts [6]. Given the extensive scale and prolonged duration of the COVID-19 pandemic [47, 48], the importance of governance effectiveness in pandemic control has become evident. The United Nations (UN) has emphasized the notion that “No one is truly safe until everyone is safe” [49].

Out of the 196 countries that reported their SPAR capacity, this study analyzed only 80 countries, with a majority falling into the category of good governance [34]. This suggests that there are still numerous countries with limited resources and inadequate governance that fail to recognize the impact of pandemic threats and struggle to control them, thereby presenting a concealed danger and hindrance to global pandemic control efforts. This factor is also suspected to contribute to the prolonged duration of the COVID-19 pandemic.

Given that the balance between policies varies across countries [44] and that good governance is crucial for building resilience during health crises, future SPAR assessments should incorporate social elements to help countries strengthen their preparedness for pandemics and other emergencies. One way to achieve this is by encouraging pre-pandemic social discussions that address the prioritization of health measures alongside other societal needs. These discussions, conducted before a crisis occurs, can help establish a more balanced approach to public health policies, ensuring that government responses align with societal values and expectations.

Social discussion refers to the exchange of ideas among individuals or groups and plays a pivotal role in shaping public opinion, fostering cooperation, and promoting understanding within a society. In the context of pandemic preparedness, structured public engagement can help governments navigate the trade-offs between different policy choices—such as balancing disease control measures with economic stability and personal freedoms. Given that pandemic control policies inherently involve competing values [44], it is essential for governments to proactively engage the public in these conversations rather than making unilateral decisions. Policies shaped through broad societal consensus are more likely to be accepted and effectively implemented, ultimately enhancing national resilience. Such decisions should reflect broader societal consensus rather than being solely determined by experts.

Our study suggests that the inaccuracy of SPAR may stem from its limited consideration of social resilience. While Article 2 of the IHR emphasizes balancing health security with economic and social stability, SPAR indicators primarily assess technical and institutional capacities. To address this gap, we propose a new SPAR indicator under Capacity 10 (Risk Communication and Community Engagement), titled “Social Discussion for Policy Balance,” which would assess whether countries proactively engage the public in priority-setting for pandemic control.

This recommendation is particularly relevant to ongoing IHR amendments, which largely focus on verifying national reports rather than expanding resilience assessment criteria. By incorporating pre-pandemic social value communication into the SPAR framework, countries could systematically strengthen public trust and social cohesion—critical components of effective pandemic response.

By addressing this gap, the revised IHR could provide a more comprehensive approach to pandemic preparedness, ensuring that future health security efforts consider not only epidemiological and healthcare capacities but also the broader societal impacts of public health interventions.

## Conclusion

The study’s findings indicate that the inaccuracy of SPAR scores is attributable to a narrow focus on disease-related measurements. By solely considering these aspects, the broader social perspective is overlooked. However, when analyzed from a social standpoint, it becomes apparent that the SPAR scores of countries effectively reflect their resilience through the quality of life experienced by their populations. Consequently, the study concludes that the limited incorporation of social aspects within the SPAR framework contributes to the inaccurate prediction of COVID-19 control outcomes.

Since the implementation of the IHR (2005), no outbreak or pandemic—including H1N1 (2009), Ebola (2014–2016), and Zika (2015–2016)—has matched the scale or duration of COVID-19, which lasted for several years. The COVID-19 pandemic was declared in March 2020 and officially ended in May 2023. As a result, SPAR scores have been assessed from a relatively narrow perspective during the pandemic.

Furthermore, the mental and social health impacts of diseases often emerge more gradually than their physical health effects. Given the ongoing discussions on revising the IHR, it is essential to incorporate social aspects into the IHR indicators, particularly those reported through the SPAR system. Enhancing SPAR with social resilience measures would improve its ability to assess countries’ capacities to manage major public health threats and better predict pandemic control outcomes.

### Limitations

This study is subject to several limitations, which are as follows. First, the analysis encompassed a limited set of 80 countries owing to a lack of complete data, potentially diminishing the overall significance of this ecological study. Additionally, it is important to acknowledge that variations in the exact timing of countries’ vaccine coverage reporting may introduce potential challenges to data quality. Additionally, measurement errors in COVID-19 status metrics due to underreporting or under ascertainment [50] and other possible unmeasured confounding factors may result in the impact of COVID-19 being underestimated.

Regarding the components used in calculating the CRS, “excessive deaths” was not used in this study due to the concern of limited data and the challenge of its technical barriers [51]. Instead, we used the 3-month case fatality rate (CFR) as one of its components for measuring COVID Status with the further intention of aligning our approach with the Bloomberg version. However, due to its significance, as revealed by the literature [51, 52], future studies may benefit from considering excessive deaths when measuring the outcome of disease control.

In this study, we acknowledge the importance of considering the reliability and validity of the data utilized. It is noteworthy that a previous study [53] has underscored certain limitations and expressed concerns regarding the data quality derived from open-source databases. Despite these acknowledged limitations, we maintained our decision to use the data. This choice is grounded in the widespread use of these resources globally and their frequent utilization as a reference for describing the situations in various countries.

Then, limitation of this study may also come from the process of transforming numeric data into binary variables. Despite the purpose, this conversion comes with certain trade-offs. One notable drawback is the potential loss of granularity and nuanced information present in the original numeric data. The binary classification may lead to a simplification of intricate patterns and variations within the dataset as well as introducing the possibility of information loss or data misrepresentation. Therefore, it is crucial to be aware when interpreting the results, as the binary representation might not capture the full spectrum of the underlying numeric values.

## Electronic supplementary material

Below is the link to the electronic supplementary material.

Supplementary Material 1

## Data Availability

No datasets were generated or analysed during the current study.

## Declarations

### Patient and public involvement

Patients and/or the public were not involved in this research.
