# Supplementary material for: Why do International Health Regulations self-assessment capacities (SPAR) scores not predict COVID-19 control outcomes? – analysis of the relationship between SPAR scores and COVID-19 resilience scores in 2021
Source: Global Health. 2025 Apr 15;21:19. doi: 10.1186/s12992-025-01111-w (PMC12001693; doi:10.1186/s12992-025-01111-w)
Supplement: Supplementary file 1 — Supplementary Material 1 [file 12992_2025_1111_MOESM1_ESM.docx]

| Table 1s. Comparison between the indicators within Bloomberg’s COVID Resilience Ranking and COVID Resilience Scores (CRS) of the countries | | |
| --- | --- | --- |
| **Indicator** | **Bloomberg’s COVID Resilience Ranking (16)** | **COVID Resilience Scores (CRS)** |
| ***Reopening Status*** | | |
| Component   - *(Source)* | People covered by vaccines   - *(Bloomberg News)* | People Covered by Vaccines   - *(Our World in Data (18))* |
|  | Lockdown severity   - (Oxford University) | Lockdown Severity   - *(Our World in Data (19))* |
|  | Flight capacity   - *(OAG)* | Travel Reopening   - *(The Humanitarian Data Exchange (HDX), Office for the Coordination of Humanitarian Affairs (OCHA) (21))* |
|  | Vaccinated travel routes   - *(Sherpa, Bloomberg News)* | Community immobility   - *(Our World in Data, Google LLC (20))* |
| ***COVID Status*** | | |
| Component   - *(Source)* | 1 -month cases per 100K   - *Johns Hopkins University* | Reproduction rate   - *Our World in Data (22)* |
|  | 3-month case fatality rates   - *Johns Hopkins University* | Case fatality rate   - *Our World in Data (22)* |
|  | Total deaths per 1M   - *Johns Hopkins University* | Test/case   - *Our World in Data (22)* |
|  | Positive test rate   - *Our World in Data* | Positive test rate   - *Our World in Data (22)* |
| ***Quality of Life*** | | |
| Component   - *(Source)* | Community mobility   - *Google LLC, Bloomberg Economics* | Happiness Index   - *World Population Review, World Happiness Report 2021 (24)* |
|  | 2021 GDP growth forecast   - *Bloomberg surveys, International Monetary Fund* | Annual GDP growth   - *The World Bank (26)* |
|  | Universal Health Coverage   - *Institute for Health Metrics and Evaluation* | Peace Index   - *Vision of Humanity (25)* |
|  | Human Development Index   - *United Nations Development Program* | Human Development Index   - *United Nations Development Program (27)* |

| Table 2s. Operational definition of COVID-Resilience Score’ measurement elements | | |
| --- | --- | --- |
| **Indicator** | **Description** | **Score** |
| ***Reopening status*** |  |  |
| *People Covered by Vaccines (18)* | - Total number of people who received at least one vaccine dose, divided by the total population of the country | More=1  Less=0 |
| *Lockdown Severity (19)* | - The stringency index, which is a composite measure based on nine response indicators including school closures, workplace closures, and travel bans, rescaled to a value from 0 to 100 (strictest). | Less=1  More=0 |
| *Travel Reopening (21)* | - Travel restrictions taken by governments by considering information including country travel restrictions, flight restrictions, the requirement of COVID-19 certificates, quarantine measures, and vaccination. | More=1  Less=0 |
| *Community immobility (20)* | - Changes in the duration of time spent in places of residence relative to a baseline day. | Less=1  More=0 |
| ***COVID status*** |  |  |
| *Reproduction Rate (22)* | - The average number of new infections caused by a single infected individual. | Low=1  High=0 |
| *Case fatality rate (22)* | - The ratio between confirmed deaths and confirmed cases. | Low=1  High=0 |
| *Test per case (22)* | - The number of tests is divided by the number of confirmed cases. | High=1  Low=0 |
| *Positive test rate (22)* | - The number of confirmed cases is divided by the number of tests expressed as a percentage. | Low=1  High=0 |
| ***Quality of life*** |  |  |
| *Happiness Index (24)* | - The happiness index in the 2021 World Happiness Report. This Report is a landmark survey of the state of global happiness that ranks 156 countries by how happy their citizens perceive themselves to be. This index ranks cities around the world by their subjective well-being and digs more deeply into how the social, urban, and natural environments combine to affect our happiness. | More=1  Less=0 |
| *Annual GDP growth (26)* | - The annual percentage growth rate of GDP at market prices is based on constant local currency from 2020 to 2021 sourced from the World Bank website. | More=1  Less=0 |
| *Peace Index (25)* | - A composite index measuring the peacefulness of countries made up of 23 quantitative and qualitative indicators each weighted on a scale of 1-5. The lower the score the more peaceful the country. We collected the data from the Global Peace Index 2021. | More=1  Less=0 |
| *Human Development Index (27)* | - An index that measures 3 key dimensions of human development; a long and healthy life, access to education, and a decent standard of living (28). We used the data from the 2021 HDI report for this study. | More=1  Less=0 |

| Table 3s. The characteristics of 80 countries | | | | | | | |
| --- | --- | --- | --- | --- | --- | --- | --- |
| **No.** | **Region** | **Reopening Status** | **COVID Status** | **Quality of Life** | **COVID Resilience Score (CRS)** | **Countries’ SPAR Scores** | **Countries’ Income Level** |
| 1. | Europe | 4 | 2 | 4 | 10 | High | HICs |
| 2. | Europe | 4 | 2 | 4 | 10 | High | HICs |
| 3. | Asia | 4 | 3 | 3 | 10 | High | HICs |
| 4. | Europe | 4 | 2 | 4 | 10 | High | HICs |
| 5. | Asia | 4 | 2 | 4 | 10 | High | HMICs |
| 6. | Europe | 4 | 2 | 3 | 9 | High | HICs |
| 7. | Europe | 4 | 2 | 3 | 9 | Low | HICs |
| 8. | Asia | 4 | 1 | 4 | 9 | High | HICs |
| 9. | Europe | 4 | 2 | 3 | 9 | High | HICs |
| 10. | Europe | 3 | 3 | 3 | 9 | High | HMICs |
| 11. | Asia | 4 | 2 | 3 | 9 | High | HICs |
| 12. | Europe | 3 | 2 | 3 | 8 | High | HMICs |
| 13. | Asia | 3 | 3 | 2 | 8 | High | HICs |
| 14. | Europe | 3 | 2 | 3 | 8 | Low | HICs |
| 15. | America | 4 | 2 | 2 | 8 | High | HICs |
| 16. | Asia | 3 | 2 | 2 | 7 | High | HICs |
| 17. | Africa | 4 | 1 | 2 | 7 | Low | HMICs |
| 18. | America | 2 | 3 | 2 | 7 | High | HICs |
| 19. | America | 2 | 2 | 3 | 7 | High | HMICs |
| 20. | Europe | 2 | 2 | 3 | 7 | High | HICs |
| 21. | Europe | 3 | 1 | 3 | 7 | Low | HICs |
| 22. | Europe | 2 | 3 | 2 | 7 | Low | HICs |
| 23. | Asia | 2 | 1 | 4 | 7 | High | HICs |
| 24. | Asia | 4 | 2 | 1 | 7 | High | HMICs |
| 25. | Asia | 4 | 0 | 3 | 7 | High | LMICs |
| 26. | Africa | 3 | 2 | 2 | 7 | Low | LICs |
| 27. | Europe | 0 | 3 | 3 | 6 | High | HICs |
| 28. | Europe | 1 | 2 | 3 | 6 | Low | HMICs |
| 29. | Africa | 2 | 2 | 2 | 6 | Low | LMICs |
| 30. | America | 1 | 2 | 3 | 6 | High | LMICs |
| 31. | Europe | 1 | 2 | 3 | 6 | High | HICs |
| 32. | Europe | 2 | 2 | 2 | 6 | High | HICs |
| 33. | Africa | 3 | 1 | 2 | 6 | Low | LICs |
| 34. | Asia | 3 | 2 | 1 | 6 | Low | LMICs |
| 35. | Europe | 2 | 2 | 2 | 6 | High | HICs |
| 36. | Africa | 2 | 1 | 3 | 6 | Low | LMICs |
| 37. | Asia | 2 | 2 | 2 | 6 | Low | LMICs |
| 38. | Europe | 2 | 2 | 2 | 6 | High | HICs |
| 39. | Africa | 4 | 1 | 1 | 6 | Low | LICs |
| 40. | Asia | 4 | 1 | 1 | 6 | Low | LMICs |
| 41. | Europe | 1 | 2 | 3 | 6 | High | HICs |
| 42. | Africa | 3 | 2 | 1 | 6 | Low | LICs |
| 43. | Europe | 2 | 2 | 2 | 6 | High | HICs |
| 44. | America | 0 | 3 | 3 | 6 | High | HICs |
| 45. | Africa | 3 | 0 | 3 | 6 | Low | LMICs |
| 46. | America | 0 | 2 | 3 | 5 | High | HMICs |
| 47. | America | 1 | 2 | 2 | 5 | Low | HMICs |
| 48. | Europe | 1 | 2 | 2 | 5 | Low | HMICs |
| 49. | Europe | 1 | 2 | 2 | 5 | Low | HICs |
| 50. | Asia | 1 | 1 | 3 | 5 | Low | HMICs |
| 51. | Asia | 2 | 1 | 2 | 5 | High | HICs |
| 52. | Africa | 2 | 1 | 2 | 5 | Low | HMICs |
| 53. | Asia | 3 | 1 | 1 | 5 | High | HMICs |
| 54. | Africa | 2 | 0 | 3 | 5 | Low | HMICs |
| 55. | Asia | 2 | 1 | 2 | 5 | Low | LMICs |
| 56. | America | 1 | 2 | 2 | 5 | High | HMICs |
| 57. | Africa | 1 | 1 | 3 | 5 | Low | LMICs |
| 58. | America | 0 | 3 | 1 | 4 | Low | HMICs |
| 59. | Asia | 2 | 2 | 0 | 4 | Low | LMICs |
| 60. | America | 0 | 3 | 1 | 4 | High | HICs |
| 61. | Europe | 1 | 1 | 2 | 4 | High | HICs |
| 62. | America | 2 | 1 | 1 | 4 | Low | HMICs |
| 63. | Africa | 1 | 1 | 2 | 4 | Low | LMICs |
| 64. | America | 1 | 2 | 1 | 4 | Low | HMICs |
| 65. | Africa | 1 | 1 | 2 | 4 | Low | LMICs |
| 66. | Europe | 1 | 0 | 3 | 4 | Low | HMICs |
| 67. | Europe | 0 | 2 | 2 | 4 | Low | HICs |
| 68. | Europe | 0 | 2 | 2 | 4 | Low | HMICs |
| 69. | Europe | 1 | 0 | 3 | 4 | Low | HMICs |
| 70. | Europe | 0 | 2 | 2 | 4 | High | HICs |
| 71. | Africa | 2 | 1 | 1 | 4 | Low | LMICs |
| 72. | America | 1 | 1 | 1 | 3 | Low | LMICs |
| 73. | Asia | 2 | 1 | 0 | 3 | High | LMICs |
| 74. | America | 1 | 1 | 1 | 3 | High | HMICs |
| 75. | Asia | 2 | 0 | 1 | 3 | Low | HMICs |
| 76. | America | 0 | 2 | 1 | 3 | High | HMICs |
| 77. | Asia | 0 | 1 | 2 | 3 | Low | LMICs |
| 78. | America | 0 | 1 | 2 | 3 | Low | HMICs |
| 79. | America | 0 | 1 | 2 | 3 | Low | HMICs |
| 80. | Africa | 0 | 1 | 1 | 2 | High | HMICs |
